# BMI, Sex as Predictors of Anterolateral Thigh Free Flap Thickness for Oropharyngeal Reconstructions

**DOI:** 10.1002/lary.70166

**Published:** 2025-09-24

**Authors:** Ibrahim M. Ibrahim, Emily Clementi, Paul F. Chisolm, Sagar Vasandani, Jonathan P. Giurintano

**Affiliations:** ^1^ Georgetown University School of Medicine Washington District of Columbia USA; ^2^ Department of Otolaryngology – Head and Neck Surgery Medstar Georgetown University Hospital Washington District of Columbia USA

**Keywords:** anterolateral thigh free flap, body mass index, head and neck cancer, microvascular reconstruction, sex

## Abstract

**Objective:**

To evaluate the relationship between body mass index (BMI), sex, and anterolateral thigh free flap (ALTFF) thickness, and to determine their impact on the use of ALT and super‐thin ALT (STALFF) flaps for oral and oropharyngeal reconstruction.

**Methods:**

Ninety‐four patients who underwent ALTFF, super thin‐ALTFF (ST‐ALTFF), or radial forearm free flap (RFFF) reconstruction following resection of oral cavity and oropharyngeal squamous cell carcinoma were included. Preoperative PET/CT scans were analyzed to measure ALTFF thickness at three points along the thigh: the proximal fourth (Point A), midpoint (Point B), and distal fourth (Point C) of the line extending from the anterior superior iliac spine to the superior lateral surface of the patella. Statistical analyses included chi‐squared, *t*‐tests, ANOVA, and Pearson's correlation to assess associations between BMI, sex, and flap thickness.

**Results:**

Forty patients (42.5%) underwent ALTFF reconstruction, 18 had ST‐ALTFF (19.1%), and 36 (38.3%) received RFFF. Pearson's correlation confirmed a strong positive relationship between BMI and flap thickness at all points (*r* > 0.62). Although BMI did not differ significantly between sexes (*p* = 0.066), females had significantly thicker flaps at all measured points (*p* < 0.0001).

**Conclusion:**

BMI and sex are reliable predictors of ALTFF thickness and influence flap selection. Integrating these factors into preoperative planning optimizes patient selection for free flap type choice, minimizing intraoperative modifications and improving reconstructive outcomes.

**Level of Evidence:**

4.

## Graphical abstract

This study evaluated BMI, sex, and their influence on anterolateral thigh free flap (ALTFF) thickness for oral/oropharyngeal reconstruction. Our results showed that BMI strongly correlated with flap thickness, and women had significantly thicker flaps, despite similar BMI. These findings suggest BMI and sex have an important role in guiding flap selection to optimize outcomes and reduce perioperative adjustments.

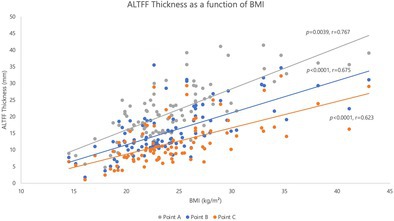

## 1 Introduction

Microvascular free tissue transfer represents the pinnacle of the reconstructive ladder for restoration of complex oropharyngeal defects following oncologic resection. The anterolateral thigh free flap (ALTFF) and radial forearm free flap (RFFF) have become workhorse flaps for head and neck soft tissue reconstructions largely due to their reliable vascularity, ease of harvest, and favorable tissue characteristics [1, 2]. Given the anatomic space limitations of the oral cavity and oropharynx, flap thickness is a crucial factor influencing flap choice. A thinner, more pliable flap is necessary for the intricate folding and contouring in the oropharynx where excessive tissue bulk may compromise functionality and aesthetic outcomes [3]. This unwanted soft tissue bulk has been the primary disadvantage of using the ALTFF for oral and pharyngeal reconstruction. Seeking to retain the low donor site morbidity of the ALTFF but avoid unnecessary soft tissue bulk, the super‐thin anterolateral thigh free flap (ST‐ALTFF) has been introduced as a feasible reconstructive option for oral and pharyngeal reconstruction [4].

Predicting ALT flap thickness can be challenging, with many surgeons relying on preoperative imaging and “pinch” testing to estimate flap thickness and guide suitability for reconstruction [5, 6]. However, such methods may be limited by the availability and variability of the measurement technique. Body mass index (BMI) is a cost‐effective and convenient metric that could guide surgeons in flap choice as a surrogate for flap thickness. Recent studies from China and Europe have explored the relationship between ALTFF thickness and BMI [7, 8]. However, there have been no recent studies to date in the United States, a country that tends to have more members of the population considered overweight or obese compared to European and Asian countries, exploring this association. Additionally, no studies have specifically included patients who had undergone ST‐ALTFFs in their evaluations.

In this study, we aim to evaluate the association between ALTFF thickness and patient demographics such as BMI and sex. We seek to provide head and neck reconstructive surgeons with a reliable and practical marker that can guide perioperative planning and improve patient outcomes.

## 2 Materials and Methods

### 2.1 Study Design and Patient Demographics

This retrospective observational study was approved by the MedStar Georgetown University Hospital IRB. Consent was waived given the retrospective nature of the study. Patients included were those who underwent ALTFF, ST‐ALTFF, and RFFF reconstruction following resection of oral cavity and oropharyngeal squamous cell carcinoma (SCCa) between 2019 and 2024. Eligible patients had preoperative skull‐base to mid‐thigh PET/CT scans available for analysis. Those who did not have PET/CT scan imaging on file were excluded. Demographic variables collected at the time of the scan included patients' age, sex, height, weight, and BMI, grouped as < 25 or > 25 kg/m^2^ (Tables 1, 2, 3).

**TABLE 1:** Influence of BMI on flap selection.

	BMI < 25	BMI > 25	*p*
Total	60 (63.8%)	34 (36.2%)	—
Age (years)	63.29	61.19	0.489
Sex			
Male	37 (61.7%)	13 (38.2%)	**0.0287** *
Female	23 (38.3%)	21 (61.8%)	
Mass (kg)	63.71	82.43	**< 0.0001** *
Height (cm)	171.69	167.03	**0.0411** *
Flap type			
ALT	34 (85%)	6 (15%)	**< 0.0001** *
ST‐ALTFF	15 (83.3%)	3 (16.7%)	
RFFF	10 (27.8%)	26 (72.2%)	
Mean ALTFF thickness (mm)			
Point A	17.38	28.65	**< 0.0001** *
Point B	12.57	21.36	**< 0.0001** *
Point C	9.6	12.76	**< 0.0001** *

*indicates statistical significant *p* < 0.05.

**TABLE 2:** Influence of sex on flap selection.

	Male	Female	*p*
Total	50 (53.2%)	44 (46.8%)	—
Age	64.33	60.48	0.184
BMI (kg/m^2^)	23.46	25.46	0.066
Mass (kg)	74.12	66.39	**0.0128** *
Height (cm)	177.31	161.7	**< 0.0001** *
Flap type			
ALTFF	26 (65%)	14 (35%)	
ST‐ALTFF	16 (88.9%)	2 (11.1%)	**< 0.0001** *
RFFF	8 (32.2%)	28 (77.8%)	
Mean ALTFF thickness (mm)			
Point A	17.89	25.5	**< 0.0001** *
Point B	11.84	20.2	**< 0.0001** *
Point C	8.64	16.23	**< 0.0001** *

*indicates statistical significant *p* < 0.05.

**TABLE 3:** Patient demographics based on selected flap type.

	ALTFF	ST‐ALTFF	RFFF	*p*
Total	40 (42.6%)	18 (19.1%)	36 (38.3%)	—
Age	64.95	60	61.22	0.34
Sex				
Males	26 (65%)	16 (88.9%)	8 (32.2%)	**< 0.0001** *
Females	14 (35%)	2 (11.1%)	28 (77.8%)	
BMI (kg/m^2^)	22.41	23.56	27.02	**0.0002** *
Mass (kg)	66.16	73.68	73.73	0.052
Height (cm)	171.91	176.78	164.49	**< 0.0001** *
Mean ALTFF thickness (mm)				
Point A	17.26	20.78	26.45	**< 0.0001** *
Point B	12.11	14.21	20.58	**< 0.0001** *
Point C	9.87	8.39	16.68	**< 0.0001** *

*indicates statistical significant *p* < 0.05.

### 2.2 Thickness Measurement

**FIGURE 1:**
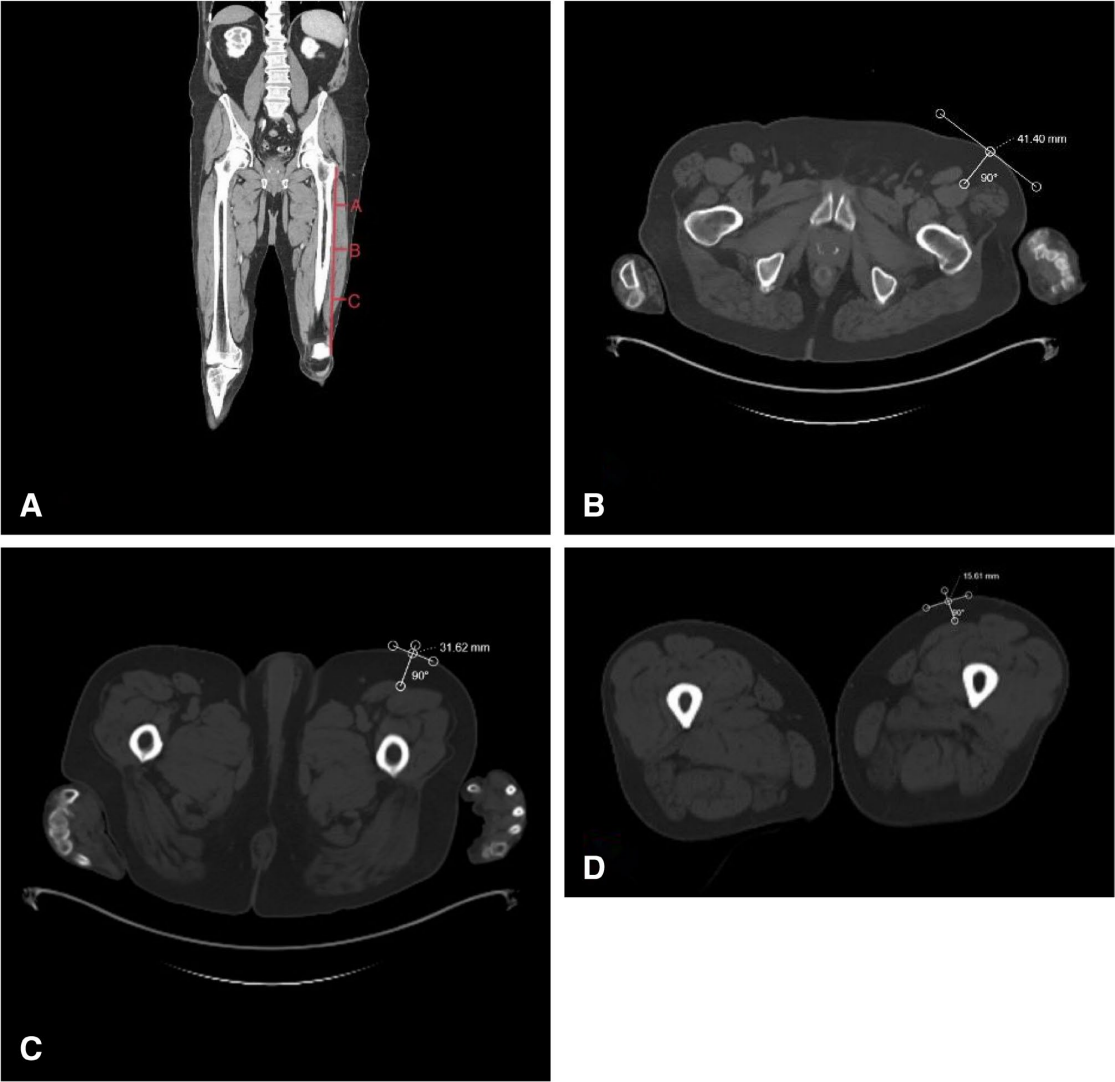
Measurement of ALTFF thickness along the thigh using a CT scan to guide harvest planning. (A) Line connecting the anterior superior iliac spine (ASIS) to the superior lateral surface of the patella. Thickness measurements were obtained at three points: (B) Point A, located at the proximal fourth of the line, (C) Point B, at the midpoint, and (D) Point C, the distal fourth. [Color figure can be viewed in the online issue, which is available at www.laryngoscope.com]

All selected patients had undergone skull‐base to mid‐thigh PET/CT scans prior to the surgery for oncologic staging. Access to the scans was through individual patient electronic medical records using ImageViewer V2 of Cerner PowerChart. Images were obtained in the supine position. ALTFF thickness and measurement methodology were defined and adopted from the approach described by Yin et al. [8] and Hsu et al. [9] Thickness was defined as the length of the perpendicular line connecting the superficial‐most point of the intersection of the rectus femoris and vastus lateralis to the overlying skin surface. Regardless of flap utilization, distances were obtained from cross‐sectional images of the thigh. Given the three‐dimensional nature of the ALTFF and the variable subcutaneous fat deposition along the length of the thigh, thickness measurements were taken at three discrete points along the thigh. The points represented different longitudinal positions along the line connecting the ASIS to the superior lateral surface of the patella, a line typically used during surgical planning and harvest of the ALTFF (Figure 1A). The three points were at the proximal 4th (Point A), midpoint (Point B), and distal 4th (Point C) of that line (Figure 1B–D).

### 2.3 Statistical Analysis

Chi‐square analysis was used to test for sex differences and flap type use frequency amongst study participants. Student's t‐test was used to compare flap thickness averages between males and females and between the BMI > 25 and BMI < 25 groups at each point. ANOVA testing was used to evaluate for differences amongst flap type groups. Pearson's correlation test was used to define the relationship between BMI and flap thickness. Excel (Microsoft Corporation 2021) and R software (v4.4.0; R Core Team 2024) were used for statistical analysis. A *p* value < 0.05 was deemed statistically significant.

## 3 Results

### 3.1 Demographics

A total of 94 patients met the inclusion criteria, 50 males (53.2%) and 44 female patients (46.8%). The average BMI was 24.4 ± 5.15 kg/m^2^ with a mean height of 170 ± 10.47 cm and a mean mass of 70.5 ± 14.97 kg. Sixty (63.8%, 37 males, 23 females) patients had a BMI < 25, whereas 34 (31.2%, 13 males, 21 females) had a BMI > 25. There was no significant difference in the BMI of males and females (*p* = 0.066).

### 3.2 Flap Selection

Within our cohort, 40 (42.6%) patients underwent ALTFF, 18 (19.1%) received ST‐ALTFF, and 36 (38.3%) had RFFF reconstruction. Overall flap thickness was highest at Point A (mean: 21.45 ± 8.28 mm) and decreased distally down the thigh (Point B 15.75 ± 7.35 mm, Point C 12.19 ± 6.55 mm; *p* < 0.0001 between Points A and B, *p* = 0.00057 between Points B and C).

### 3.3 Influence of BMI on Flap Type and Thickness

The majority of patients who underwent ALTFF and ST‐ALTFF reconstructions had a BMI < 25; 34 (85%) and 15 (83.3%), respectively. Conversely, most of the patients who received RFFF reconstruction had a BMI > 25 (26, 72.2%). There was no difference in BMI (*p* = 0.259) or ALTFF thickness measurements (Point A *p* = 0.065, Point B *p* = 0.16, Point C *p* = 0.122) between patients who underwent ALTFF and ST‐ALTFF reconstruction. Those who received RFFF were found to have a higher average BMI compared to both the ALTFF (*p* = 0.00025) and ST‐ALTFF groups (*p* = 0.011). The RFFF group was also found to have higher ALTFF thickness measurements at all points compared to the ALTFF group (Point A *p* < 0.0001, Point B *p* < 0.0001, Point C *p* < 0.0001) and the ST‐ALTFF group (Point A *p* = 0.012, Point B *p* = 0.0013, Point C *p* < 0.0001).

**FIGURE 2:**
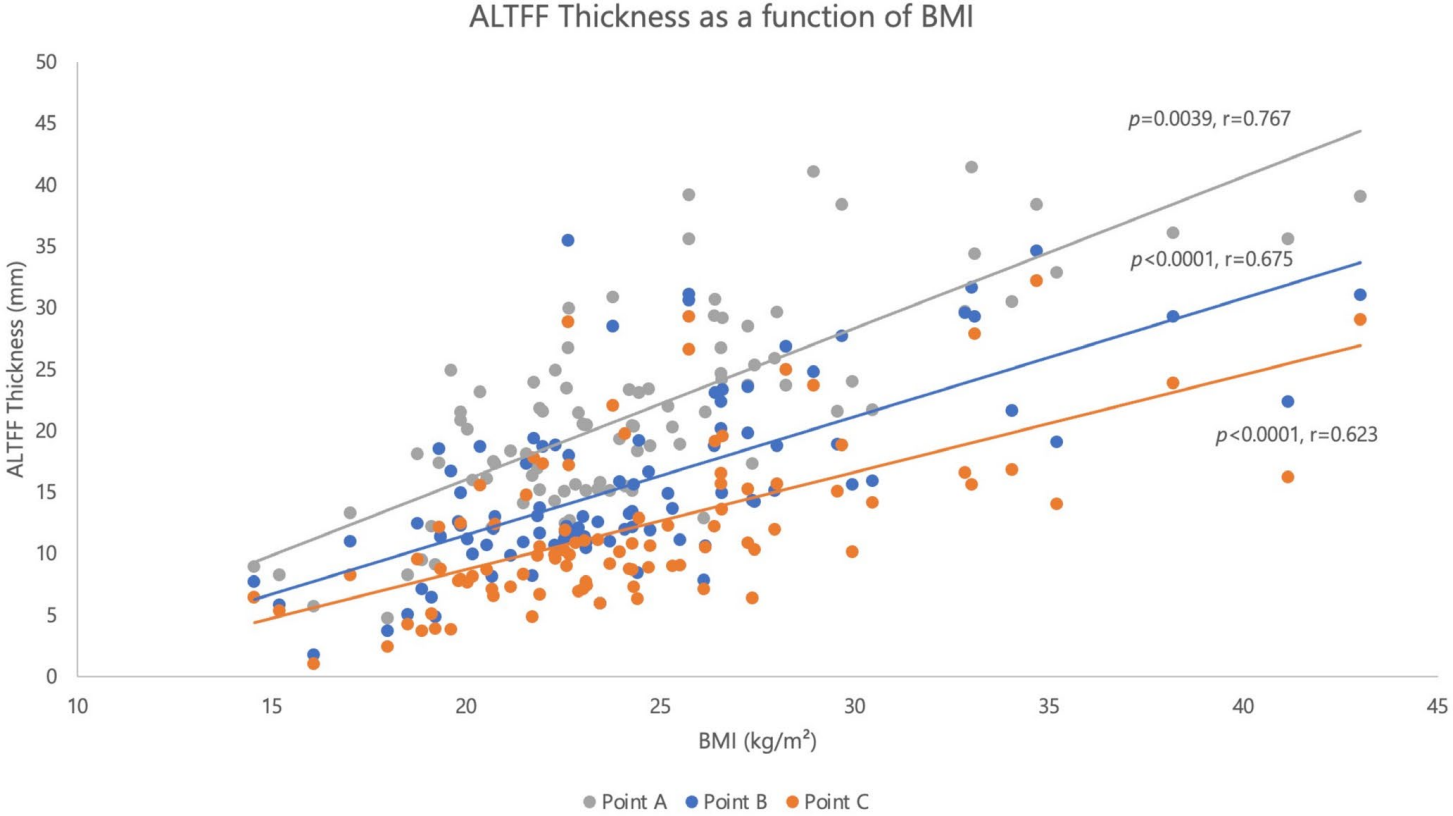
Flap thickness as a function of BMI. [Color figure can be viewed in the online issue, which is available at www.laryngoscope.com]

When comparing the different BMI groups, the higher BMI group was found to have larger average thickness at each measurement point (Point A, *p* < 0.0001; Point B, *p* < 0.0001; Point C, *p* < 0.0001). The influence of BMI on flap selection is presented in Table 1. There was a statistically significant correlation between BMI and ALTFF thickness at each point (Point A: *r* = 0.767, *p* = 0.0039; B: *r* = 0.675, *p* < 0.001; C: *r* = 0.623, *p* < 0.001, Figure 2).

### 3.4 Influence of Sex on Flap Type and Thickness

Twenty‐six of the 40 patients who underwent ALTFF reconstruction were male (65%). Most patients who underwent RFFF were female (28), accounting for 77.8%. Of the 18 patients who underwent ST‐ALFF, the majority were male (89.5%). Female patients had thicker measurements at each point when compared to male patients (Point A: *p* < 0.0001; B: *p* < 0.0001; C: *p* < 0.0001), despite there not being a statistically significant difference in BMI between males and females in our cohort (Table 2). More detailed characteristics of patient groups based on flap type utilized are presented in Table 3.

## 4 Discussion

Arguably one of the most important decisions a reconstruction surgeon will make is the choice of the free flap donor site, and determining the optimal free flap can present a significant pre‐operative challenge. In the historical dichotomy of traditional ALTFF versus RFFF for soft tissue reconstruction, surgeons have often relied on heuristics such as the “pinch test” to estimate thigh flap thickness. Although this method is quick and cost‐effective, it is inherently subjective and depends largely on the surgeon's gestalt and experience. In the search for a thin, pliable flap that avoids the donor‐site morbidity of the RFFF, other options such as the lateral arm, superficial iliac circumflex artery perforator (SCIP), and the relatively novel medial sural artery perforator (MSAP) flaps have been explored [10, 11, 12, 13]. Although the MSAP has been shown to provide a pliant flap even in patients with higher BMI, many reconstructive surgeons did not receive training on MSAP harvest in the course of their training. Our group has previously demonstrated the feasibility of the ST‐ALTFF in oral cavity and oropharyngeal reconstruction, capitalizing on an easily adaptable technical derivation to the widely adopted traditional ALTFF [4]. However, even in these cases, patient selection was guided by a single surgeon's “pinch test,” which could potentially limit the reproducibility of our results at other institutions. For this reason, a more objective measure in pre‐operative assessment for determining different types of flap candidacy is imperative.

BMI has been previously explored as a low‐cost, objective adjunct to predict thigh thickness in patients undergoing microvascular reconstruction. Some studies suggest there is not a significant relationship between pre‐operative BMI [14], whereas others have demonstrated a positive correlation between BMI and thigh thickness [7, 15]. Notably, these studies were conducted in international populations, potentially limiting their external validity, especially in the context of the United States' obesity epidemic. In our patient cohort, the observed average BMI of all patients was 24.4 kg/m^2^, which is lower than the average US BMI of 29.2 kg/m^2^ according to the National Health and Nutrition Examination Survey [16]. This disparity likely reflects the demographics of the greater DC area, compounded by the catabolic effects of cancer on patients' nutritional status. Importantly, despite this relatively lower BMI range, we found a strong positive correlation between BMI and ALTFF thickness at all measured points on the thigh. Patients with higher BMI had proportionally thicker ALT flaps along the entire length of the thigh, reinforcing the existing body of literature that supports BMI as a reliable predictor of flap thickness.

Although BMI was strongly associated with flap thickness, our data also highlight sex‐based differences in thigh adiposity that BMI alone does not capture. We observed that female patients had significantly thicker ALTFF thicknesses than males at all measured points, despite similar BMI distributions between sexes in our cohort. This finding has been demonstrated in prior articles showing that women tend to have thicker subcutaneous thigh tissue than men of comparable BMI [5, 7, 8]. Yin et al. [5] noted that female patients had significantly greater ALT thickness than males at multiple thigh locations, even when stratified by BMI groups. This sex disparity can be explained by well‐known patterns of fat distribution: Estrogen drives women to accumulate more subcutaneous fat in the gluteofemoral region, whereas men tend to carry more visceral fat intra‐abdominally [17]. Thus, even at equivalent BMI, women frequently have thicker ALT flaps than men, limiting their suitability in oral and oropharyngeal reconstruction.

This anatomical difference between sexes was reflected in flap selection patterns across our cohort. Most of the ALTFFs and ST‐ALTFFs in our series were performed in male patients, whereas most patients undergoing an RFFF reconstruction were female. Although there was no statistically significant difference in BMI between sexes, both the ALTFF and ST‐ALTFF groups had a significantly lower mean BMI than the RFFF group, a finding attributable to the combined influence of sex and BMI on thigh thickness. Specifically, despite comparable BMIs, female patients demonstrated greater subcutaneous adiposity and therefore composed most of those who underwent RFFF reconstruction. In contrast, male patients with lower BMIs consistently presented with favorable thigh thickness, making the ALTFF and ST‐ALTFF preferable. Although most of the ST‐ALTFF patients in our series had a lower BMI, we show that it is a versatile reconstructive option that can be used reliably across the BMI spectrum. Notably, there were exceptions to general trends; some female patients or those with elevated BMIs still underwent ALTFF reconstruction, and conversely, a subset of RFFF patients were male and/or had lower BMIs. Ultimately, the choice between an ALT and RFFF often depends on surgeon preference and experience, particularly with flap thinning and intraoperative debulking techniques. Our observed patterns underscore the importance of considering both BMI and sex during preoperative assessment, as their interplay may directly inform flap suitability.

Our results also have important implications in the current era of virtual preoperative consultations, where physical examination of donor sites is often limited. Given that BMI and sex are readily available demographic variables, they can serve as practical, objective predictors of flap thickness during virtual evaluations. This allows reconstructive surgeons to anticipate flap characteristics and consider super‐thin harvesting techniques or alternative donor sites when appropriate. Integrating these predictors into preoperative planning can optimize flap selection and improve overall reconstructive efficiency in remote or resource‐constrained settings.

## 5 Limitations

This study has several limitations. A key limitation is the subjective nature of flap selection, which was based on individual surgeon judgment and the subjective pinch test, rather than standardized criteria. As a result, patients with thicker thighs on physical exam may have been preferentially selected for alternative flaps, introducing potential selection bias. Furthermore, ALT flap thickness was assessed using PET/CT imaging alone, without intraoperative measurements, which limits direct validation of the radiographic estimates. Moreover, as a retrospective, single‐institution study, the findings may not be broadly generalizable, underscoring the need for prospective research in larger and more diverse populations to further validate these observations.

## 6 Conclusion

BMI and sex serve as practical, objective, and cost‐effective screening tools for estimating the feasibility of the ALTFF harvest in oropharyngeal reconstruction. The strong correlation between BMI and flap thickness supports its role in guiding preoperative decision‐making, allowing surgeons to anticipate whether a primary ALTFF is achievable or if an alternative approach, such as flap thinning or use of a different donor site, may be required. However, BMI alone does not fully capture differences in thigh adiposity, as our findings demonstrate that female patients have significantly thicker thigh tissue at equivalent BMIs. Incorporating BMI, alongside patient sex, into preoperative planning facilitates a strategically individualized reconstructive strategy and may reduce reliance on costly imaging or subjective bedside assessments. This information may be especially useful in the modern era of virtual pre‐operative visits and can guide surgeons' decision making. Overall, these findings support the integration of BMI and sex into routine preoperative evaluation to guide the selection of appropriate candidates for the different routinely used free flap types, minimize intraoperative modifications, and optimize reconstructive outcomes.

## Conflicts of Interest

Dr. Giurintano receives material research support from Ambu Inc. The other authors declare no conflicts of interest.

## Data Availability

The data that support the findings of this study are available from the corresponding author upon reasonable request.
